# *Euastacus morgani* sp. n., a new spiny crayfish (Crustacea, Decapoda, Parastacidae) from the highland rainforests of eastern New South Wales, Australia

**DOI:** 10.3897/zookeys.85.1237

**Published:** 2011-03-11

**Authors:** Jason Coughran, Robert B. McCormack

**Affiliations:** 1 Environmental Futures Centre, Griffith School of Environment, Gold Coast Campus, Griffith University, Queensland, Australia, 4222; 2 Australian Crayfish Project, c/- Australian Aquatic Biological Pty Ltd, P.O. Box 3, Karuah, NSW, Australia, 2324

**Keywords:** *Euastacus*, Parastacidae, montane, rainforest, spiny crayfish, Australia

## Abstract

Euastacus morgani sp. n., is described from a highland, rainforest site in Bindarri National Park, in eastern New South Wales, Australia. Euastacus morgani is found living sympatrically with two more common species, Euastacus dangadi Morgan, 1997 and Euastacus neohirsutus Riek, 1956. Systematically, the species belongs in the ‘*simplex*’ complex of the genus that includes Euastacus simplex Riek, 1956, Euastacus clarkae Morgan, 1997, Euastacus maccai McCormack and Coughran 2008 and E. morgani. This new species differs from its nearest congenor, Euastacus simplex, in having three mesial carpal spines. A key to the ‘*simplex*’ complex is presented.

## Introduction

The Australian spiny crayfish genus Euastacus Clark, 1936 is the most species-rich of all genera in the family Parastacidae, the southern hemisphere freshwater crayfishes. Clark (1936) erected the genus based on the type species Cancer serratus Shaw, 1794, a junior homonym later corrected to Astacoides spinifer Heller, 1865 (see Morgan 1986). Although Clark (1936) initially recognized six species, subsequent work would clarify that there were seven species contained within the material at that time, each of which had been recognized by a different authority at varying times over the preceding century. Clark (1941) also revised the genus, in the process describing two new species. Notable taxonomic contributions were later made by Riek (1951, 1956, 1969), who described 14 of the currently recognized species, and shortly thereafter a single species was described from far north Queensland by Monroe (1977). More recently, a revision of the genus was undertaken by Morgan (1983, 1986, 1988, 1989, 1997), whose comprehensive works resulted in the description of 17 new species, and was followed by a dramatic increase in research on the taxonomy, biology and ecology of the genus. Since then, eight additional species have been discovered and described, all from restricted highland areas of eastern and northern Australia (Short and Davie 1993; Coughran 2002, 2005; Coughran and Leckie 2007; McCormack and Coughran 2008).

In this paper, we describe a further new species from a highland, rainforest site in central eastern Australia. In 2003, a single specimen of this new species of Euastacus was discovered during a field survey in Bindarri National Park near Lowanna, New South Wales. Although photographs were taken, the specimen itself was subsequently lost. After many attempts to obtain further specimens from the original site and in the surrounding area, survey efforts finally yielded new specimens in 2008. We now present a formal description for Euastacus morgani sp. n.

## Methods

Morphological terminology, character descriptions, size references and ratios follow those described by Morgan (1986). Orbital or ocular carapace length (OCL) is the standard index measurement for this genus, and extends obliquely from the eye socket to the dorsal posterior of the carapace (Morgan 1986). Specimens are deposited in the

collections of the Australian Museum (AM) and the Australian Crayfish Project (ACP).

## Taxonomy

### Euastacus morgani sp. n.

urn:lsid:zoobank.org:act:07E283DB-82D9-45CB-9CD0-9BBC4E80FE3C

Figures 1 2

#### Material Examined.

HOLOTYPE. AM P.84263, Australia, New South Wales, tributary of Little Nymboida River, upstream of Eastern Dorrigo Way road crossing near Lowanna, on periphery of Bindarri National Park, rainforest, 30.2288°S, 152.9203°E, 25 March 2008, J. Coughran and R. B. McCormack, 30.88 mm OCL, female. PARATYPES. AM P.84264–P.84266, type locality, 25 March 2008, J. Coughran and R. B. McCormack, 29.94–39.87 mm OCL, 2 females, 1 male.

#### Other Material.

ACP 1101–1105, type locality, 25 March 2008, J. Coughran and R. B. McCormack, 22.41–31.65 mm OCL, 3 females, 2 males. ACP 1095–1096, type locality, 25 March 2008, J. Coughran and R. B. McCormack, 17.17–19.77 mm OCL, 1 male, 1 female. ACP 1098, type locality, 25 March 2008, J. Coughran and R. B. McCormack, 14.85 mm OCL, 1 male.

#### Diagnosis.

Male cuticle partition present. Rostrum with 3 or 4 small marginal spines per side, extending to midlength. Suborbital spine of medium size. Thorax with 10–20 very small to small thoracic spines per side. Abdominal somites with tiny to small, moderately sharp to sharp Li and Lii spines. D-L and D abdominal spines present on somites 2–3 of some large specimens. Apical propodal spines absent. Ventral lateral propodal spine row moderately developed, usually with 4–5 centrally located spines. Spines above propodal cutting edge absent. Dactylus with 1 apical mesial spine and 1 dorsal mesial basal spine. Marginal dactylar basal spines absent. Dactylar finger with 1 or 2 tiny apical spines above cutting edge. Carpus with 3 mesial and 2 lateral spines. Ventromesial carpal spines smaller than ventral carpal spine. Setation light.

**Figure 1.**
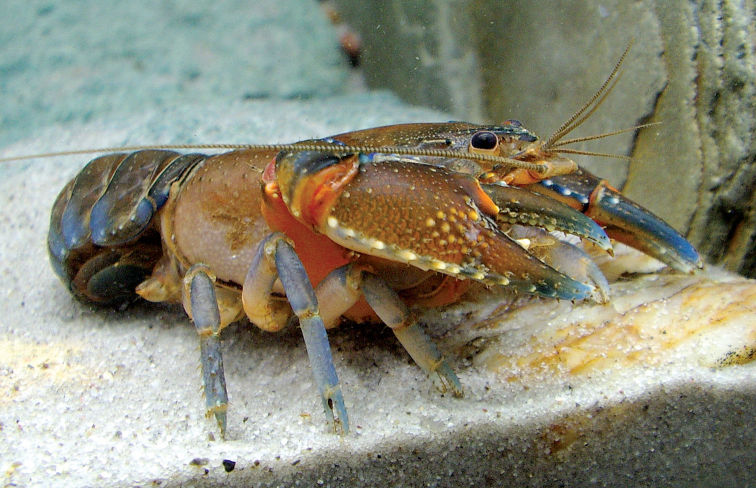
Euastacus morgani sp. n. Female specimen, ACP 1103.

**Figure 2.**
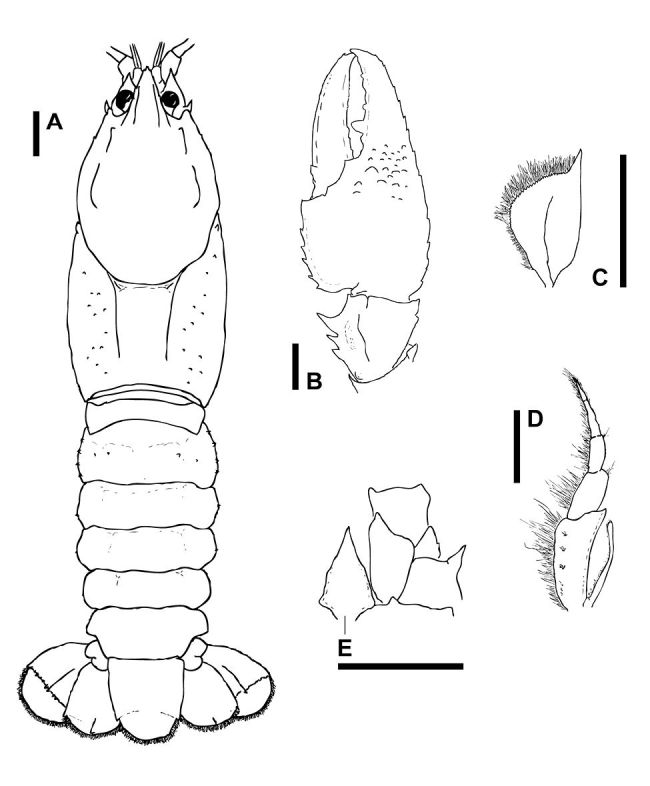
Euastacus morgani sp. n. Dorsal view of body **A** and cheliped **B**, holotype specimen, AM P.84263. Dorsal view of antennal squame **C**, lateral view of third maxilliped **D** and ventral view of left hand side of cephalon showing interantennal scale **E**, specimen ACP 1103. Scale bars = 5 mm.

#### Size.

The maximum size of specimens collected was 39.9 mm OCL.

#### Description.

##### Rostrum.

Rostrum short, usually reaching base of third antennal segment (occasionally not quite reaching base, and rarely extending as far as midlength of segment). Rostral sides slightly convergent to convergent, rostral bases divergent. Rostrum with 3 or 4 small marginal spines, extending to approximately rostral midlength, or beyond. Acumen similar in size to marginal spines. OCL/Carapace Length: 0.83–0.89. Rostrum Width/OCL: 0.16–0.19.

##### Cephalon.

Numerous bumps and protrusions in cephalic region, with 1 (occasionally 2) on each side developed into more prominent, but small and blunt cephalic spine. First post-orbital ridge spine distinct but small. Second post-orbital ridge spine barely discernible to small; posterior to spine each ridge poorly developed initially, with large and swollen bump at posterior end. Suborbital spine medium in size, and acute.

##### Antenna.

Basipodite spines small to medium. Coxopodite without distinct spines but with large, rounded development of coxopodal plate at mesial end. Interantennal spine of medium width and usually with slightly scalloped margins. Antennal squame lacking marginal spines, with near triangular inflation at midlength. Scale Length/OCL: 0.09–0.12.

##### Thorax.

Thorax with 1–3 barely discernible to small cervical spines on each side, flattened and triangular in shape with moderate to sharp point. Dorsal thoracic spines present and numbering 10–20 on each side. Dorsal thoracic spines very small to small but distinct, and larger than general tubercles; spines highlighted against the carapace by profile (raised, but blunt) and/or colour (yellow-brown, grey-brown, grey or blue-grey). General tubercles small to medium in size (very small on specimens <25 mm OCL), and of moderate density. Areola Length/OCL: 0.35–0.39. Areola Width/OCL: 0.16–0.21. Carapace Width/OCL: 0.53–0.62. Carapace Depth/OCL: 0.44–0.50.

##### Sternal Keel.

Lateral processes to first pereiopods (LPr1) slightly apart, slightly open to open in orientation, and with semi-abrupt posterior margins. LPr2 apart and with slightly open to open orientation. LPr3 narrow, and LPr4 of medium width. LPr2 to LPr4 all with distinctive, sharply defined margins, and sternal keel between both LPr2–LPr3 and LPr3–LPr4 entire and sharply developed.

##### Abdomen.

Abdominal somite 1 lacking spines. Somite 2 with 3–5 tiny to small, moderately sharp to sharp Li spines. Somites 3–6 with 1 Li spine, decreasing in size posteriorly to be barely discernible on somite 6 (absent on some specimens <25 mm OCL). Frequently 1 or 2 small Lii spines on somites 3–6 of specimens > 20 mm OCL. Abdominal D-L spines present on somites 2–3 of one specimen, and on somites 2–6 of the largest specimen. Abdominal D spines also present on somites 2–3 of these two specimens only. Abdomen Width/OCL: 0.48–0.54 (male), 0.50–0.55 (female). OCL/Total Length: 0.39–0.43.

##### Tailfan.

Standard tailfan spines small. Telson and uropods lacking surface and marginal spines. Telson Length/OCL: 0.33–0.37.

##### First Cheliped.

Chelae usually intermediate in shape, occasionally elongate or stout.

##### Propodus.

Dorsal lateral propodal spine row extending from apex to base of propodus. Ventral lateral propodal spine row less developed, with 1–6 (usually 4 or 5) more or less centrally located spines. Numerous bumps and protrusions lateral to dactylar base dorsally, of which 1 or 2 are usually distinctly larger. Bumps and protrusions also present on ventral surface of propodus lateral to dactylar base, often including 1–4 more prominently developed spines extending along propodal finger. Propodus with 5–7 mesial spines. Dorsal apical propodal spines absent. Two blunt spines at dactylar articulation. Propodal surface posterior to dactylar articulation lacking spines, with 2 or 3 distinctly deep punctuations. Pre-carpal area lacking spines, with occasional distinct punctuations. Spines above propodal cutting edge absent on both dorsal and ventral surfaces (2 barely discernible apical spines on dorsal surface of one specimen ACP1099). Prominent tooth near dactylar articulation. Propodus Length/OCL: 0.81–0.90 (male), 0.80–0.94 (female). Propodus Width/PropL:0.46–0.50. Propodus Depth/Propodus Length: 0.26–0.31.

##### Dactylus.

Usually 1 or 2 barely discernible to small apical spines above dactylar cutting edge (absent on one specimen). Spines above cutting edge absent on ventral surface. Dactylus with 1 apical mesial spine and 1 dorsal mesial basal spine. All other dactylar spines absent (one regenerate chela with a small marginal dactylar basal spine). Dactylar groove distinct. Dactylus Length/Propodus Length: 0.55–0.61.

##### Carpus.

Carpus with 3 mesial spines, decreasing in size posteriorly; generally produced to sharp point. Lateral margin of carpus with 2 spines. Ventral surface of carpus with large, sharp ventral spine and 1 or 2 tiny to medium, blunt ventromesial spines. Dorsal surface with deep groove; bearing 1–3 large, bluish-green, blunt bumps or spines (occasionally merging to effectively form raised ridge or boss) on dorsal surface mesial to groove.

##### Merus.

Merus with 6 or 7 small to medium dorsal spines, and a barely discernible or small distolateral spine.

##### Third Maxilliped.

Laterodistal corner of ischium produced to a distinct point. Exopodite shorter than or about as long as ischium (average 0.93 × ischium length).

##### Gastric Mill.

Gastric mills were carefully extracted and examined for three specimens ranging in size from 29.94 to 39.87 mm OCL. Zygocardiac ossicle with 1.0–1.5 teeth anterior to ossicle ear (TAA), and 4.0–4.5 teeth anterior to posterior margin of ear (TAP), with tooth spread of 2.5–3.0. Urocardiac ossicle with 6 or 7 ridges.

##### Setation and Punctation.

Setae sparse and short. Sparse to moderate and fine punctation on body. Chelae with occasional deep punctuations.

##### Colouration.

Dorsally brown or green-brown with pale cream cephalic and cervical spines. Thoracic spines usually distinct from thorax background, and varying in colour (cream, grey, grey-brown or blue-grey). Abdominal somites marked heavily with blue laterally, with cream Li spines. First chelae mesially blue, with cream-tipped mesial propodal spines. Dorsal carpal bumps blue (often forming a raised, blue ridge). Walking legs blue-grey. Lateral propodal spines cream. Ventrally, a varying wash of pale blue-green, pink and orange, with a dull grey abdomen and tailfan. Merus of first cheliped and antennal bases vivid orange.

#### Sexes.

Males with cuticle partition. Female specimens without fully mature gonopores. All female specimens have calcified gonopores that lack marginal setation. Gonopores of four largest female specimens (29.9–39.9 mm OCL) with slightly incised rims, those of largest specimen appear to be in state of decalcification, being partially membranous on mesial margins. Thus, it would seem that female maturity occurs close to 40 mm OCL.

#### Etymology.

Named to honour Gary J. Morgan, whose landmark research on the Australian spiny crayfish genus Euastacus (Morgan 1983, 1986, 1988, 1989, 1991, 1997) has been pivotal to our understanding of these animals.

## Discussion

**Figure 3.**
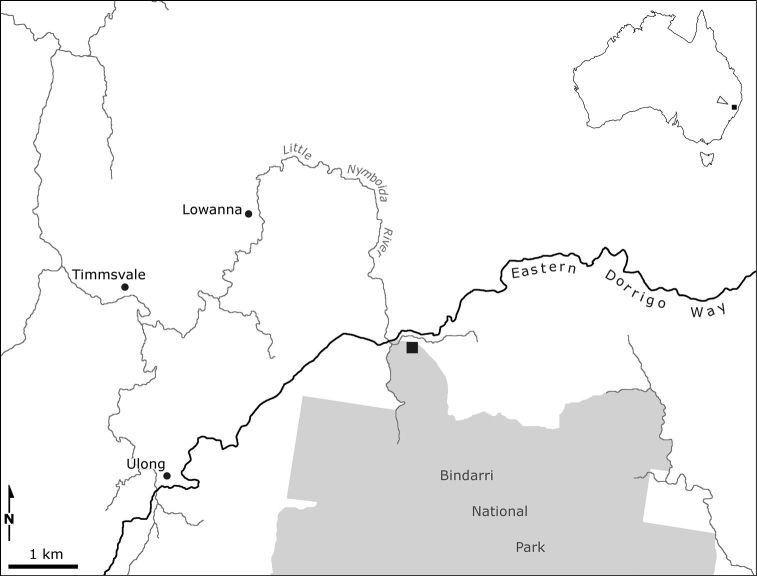
Location of the type locality (black square) on the northern margin of Bindarri National Park, in central eastern Australia.

Euastacus morgani sp. n. is known only from the type locality, a highland (~600 m a.s.l.), rainforest site at the northern margin of Bindarri National Park, in a tributary to the Little Nymboida River (Figure 3). Two comparatively widespread species of Euastacus occur in the area, including a site in Little Nymboida River proximal to the type locality, Euastacus dangadi Morgan, 1997 and Euastacus neohirsutus Riek, 1956. We have surveyed numerous sites in the area, but our efforts yielded only these two more common species.

The species constructs a more or less horizontal burrow entrance from the water’s edge back into the stream banks. The specimens were collected by probing these bank burrow systems by hand until the crayfish attempted to exit via another entrance hole, typically located 1–2 m further back from the creek on the adjacent forest floor. The successful sampling was undertaken at night.

Although the precise locality is uncertain, Gary Morgan collected juvenile Euastacus specimens at a nearby site (‘Brimbin Creek’, near Lowanna) that were too small for taxonomic determination, although they appeared to be different to all local species (Morgan 1983). Morgan (1983) collected these specimens in sympatry with Euastacus neohirsutus, and observed differences to that species on the following taxonomic features: larger rostral carinae; larger antennal basipodite spine; better developed ventral lateral propodal spine row of the cheliped; lacking dorsal apical propodal spines; lacking spines above the cutting edge of the propodus. All of these characters are also true of Euastacus morgani, and thus it is likely that the small specimens observed by Gary Morgan were juveniles of this new species.

Euastacus morgani appears most similar in morphology to Euastacus simplex Riek, 1956. It can be distinguished among other characters by having: 3 instead of 2 mesial carpal spines on the cheliped; no spines above the propodal cutting edge of the cheliped; medium instead of barely discernible or small suborbital spines; poor development of the ventral lateral propodal spine row; a proportionally shorter interantennal scale; and a proportionally shorter exopodite and more pointed anterior terminus to the ischium of the third maxilliped. Systematically, the species thus fits within the ‘*simplex*’ complex (Morgan 1997), which includes Euastacus simplex, Euastacus clarkae Morgan, 1997, Euastacus maccai McCormack and Coughran, 2008, and Euastacus morgani. A key is provided below to distinguish Euastacus morgani from other species in the ‘*simplex*’ complex.

The description of Euastacus morgani increases the number of described species in this charismatic genus to 50 (Table 1). There are further taxa still awaiting formal description, and large areas of potentially suitable habitat that have yet to be surveyed. Unfortunately, 80% of Euastacus are considered to be threatened (Coughran and Furse 2010; Furse and Coughran in press a, b, c; IUCN 2010), and there is thus a sense of urgency for continued field surveys and taxonomic work on this genus. It would appear that Euastacus morgani has a severely restricted, highland distribution, and may be susceptible to similar threats facing other species in the genus, particularly those relating to over-collection, exotic species and climate change (Furse and Coughran in press a, b, c). Further research into its ecology is warranted.

**Table 1.** Species of Euastacus and their current conservation status listing on the IUCN Red List of Threatened Species (IUCN 2010). Conservation status is provided in parentheses, as follows: **DD** Data Deficient; **LC** Least Concern; **VU** Vulnerable; **EN** Endangered; **CR** Critically Endangered.

Euastacus armatus (von Martens, 1866) (DD)	Euastacus kershawi (Smith, 1912) (LC)
Euastacus australasiensis (H. Milne Edwards, 1837) (LC)	Euastacus maccai McCormack & Coughran, 2008 (EN)
Euastacus balanensis Morgan, 1988 (EN)	Euastacus maidae (Riek, 1956) (CR)
Euastacus bidawalus Morgan, 1986 (EN)	Euastacus mirangudjin Coughran, 2002 (CR)
Euastacus bindal Morgan, 1989 (CR)	Euastacus monteithorum Morgan, 1989 (CR)
Euastacus bispinosus Clark, 1941 (VU)	Euastacus morgani sp. n. (n/a)
Euastacus brachythorax Morgan, 1997 (EN)	Euastacus neodiversus Riek, 1969 (EN)
Euastacus clarkae Morgan, 1997 (CR)	Euastacus neohirsutus Riek, 1956 (LC)
Euastacus claytoni Riek, 1969 (EN)	Euastacus pilosus Coughran & Leckie, 2007 (EN)
Euastacus crassus Riek, 1969 (EN)	Euastacus polysetosus Riek, 1951 (EN)
Euastacus dalagarbe Coughran, 2005 (CR)	Euastacus reductus Riek, 1969 (LC)
Euastacus dangadi Morgan, 1997 (LC)	Euastacus rieki Morgan, 1997 (EN)
Euastacus dharawalus Morgan, 1997 (CR)	Euastacus robertsi Monroe, 1977 (CR)
Euastacus diversus Riek, 1969 (EN)	Euastacus setosus (Riek, 1956) (CR)
Euastacus eungella Morgan, 1988 (CR)	Euastacus simplex Riek, 1956 (VU)
Euastacus fleckeri (Watson, 1935) (EN)	Euastacus spinichelatus Morgan, 1997 (EN)
Euastacus gamilaroi Morgan, 1997 (CR)	Euastacus spinifer (Heller, 1865) (LC)
Euastacus girurmulayn Coughran, 2005 (CR)	Euastacus sulcatus Riek, 1951 (VU)
Euastacus gumar Morgan, 1997 (EN)	Euastacus suttoni Clark, 1941 (VU)
Euastacus guruhgi Coughran, 2005 (CR)	Euastacus urospinosus (Riek, 1956) (EN)
Euastacus guwinus Morgan, 1997 (CR)	Euastacus valentulus Riek, 1951 (LC)
Euastacus hirsutus (McCulloch, 1917) (EN)	Euastacus woiwuru Morgan, 1986 (NT)
Euastacus hystricosus Riek, 1951 (EN)	Euastacus yanga Morgan, 1997 (LC)
Euastacus jagabar Coughran, 2005 (CR)	Euastacus yarraensis (McCoy, 1888) (VU)
Euastacus jagara Morgan, 1988 (CR)	Euastacus yigara Short & Davie, 1993 (CR)

## Key to the ‘simplex’ complex of the genus Euastacus

**Table:** 

1	Chelae with elongate, tapered fingers. Apart from one or two large molars, development of teeth on cutting edges of chelae distinctly reduced. Gape between fingers distinctly broad and lanceolate in shape	Euastacus maccai McCormack and Coughran
–	Chelae with stout fingers, without distinctive gape between fingers. Lesser cutting edge teeth of moderate size	2
2	Cheliped with 3 mesial carpal spines	Euastacus morgani sp. n.
–	Cheliped with 2 mesial carpal spines	3
3	Dorsal apical propodal spines present. Suborbital spine medium to large	Euastacus clarkae Morgan
–	Dorsal apical propodal spines absent. Suborbital spine barely discernible to small	Euastacus simplex Riek

## Supplementary Material

XML Treatment for Euastacus morgani
